# Research on Multi-Terminal’s AC Offloading Scheme and Multi-Server’s AC Selection Scheme in IoT

**DOI:** 10.3390/e24101357

**Published:** 2022-09-24

**Authors:** Jiemei Liu, Fei Lin, Kaixu Liu, Yingxue Zhao, Jun Li

**Affiliations:** School of Information and Automation, Qilu University of Technology (Shandong Academy of Sciences), Jinan 250353, China

**Keywords:** Mobile Edge Computing (MEC), Simultaneous Wireless Information and Power Transfer (SWIPT), AC offloading algorithm, AC selection algorithm

## Abstract

Mobile Edge Computing (MEC) technology and Simultaneous Wireless Information and Power Transfer (SWIPT) technology are important ones to improve the computing rate and the sustainability of devices in the Internet of things (IoT). However, the system models of most relevant papers only considered multi-terminal, excluding multi-server. Therefore, this paper aims at the scenario of IoT with multi-terminal, multi-server and multi-relay, in which can optimize the computing rate and computing cost by using deep reinforcement learning (DRL) algorithm. Firstly, the formulas of computing rate and computing cost in proposed scenario are derived. Secondly, by introducing the modified Actor-Critic (AC) algorithm and convex optimization algorithm, we get the offloading scheme and time allocation that maximize the computing rate. Finally, the selection scheme of minimizing the computing cost is obtained by AC algorithm. The simulation results verify the theoretical analysis. The algorithm proposed in this paper not only achieves a near-optimal computing rate and computing cost while significantly reducing the program execution delay, but also makes full use of the energy collected by the SWIPT technology to improve energy utilization.

## 1. Introduction

In the 5G era, the unprecedented growth of real-time Internet of Things (IoT) applications can generate a vast volume and variety of data [1]. It not only brings great convenience to people’s lives but also challenges the demands for computing rate and energy consumption [2]. On the one hand, traditional cloud computing [3] could not meet people’s needs, and so gradually mobile gradually. Mobile edge computing (MEC) came into being. Compared with cloud computing services [4,5,6], MEC has gradually entered the market with the advantages of having a more decentralized server location deployment strategy, shorter response time, and more immediate data processing. MEC involves devices offloading part or all of their computing tasks to use larger edge servers to process the data. The technology can improve the computing efficiency and performanceand bring with it better service quality [7]. On the other hand, simultaneous wireless information and power transfer (SWIPT), a green technology that can reduce energy consumption effectively, has also attracted extensive attention. SWIPT can transmit information and energy at the same time [8]. When using SWIPT technology, we can ignore the problem of replacing the battery. At present, the edge computing area includes network operators, company production networks, smart home systems, face recognition, wearable devices, smart grids and other scenarios. In these scenarios, the data processing power of some smart phones, computers, and other application devices is limited and cannot satisfy the needs of users to complete computing tasks. Data from dozens or even hundreds of devices are sent to an edge server at the edge of the network to process data quickly and efficiently. At the same time, based on the requirements of user experience, the process of data processing should minimize energy consumption. Thus, we combine SWIPT technology and MEC technology to solve the problems of user offloading and server selection.

The main contributions of this paper are summarized as follows:

- Considering the characteristics of MEC’s data processing capacity and SWIPT’s energy collection, a multi-terminal, multi-relay, and multi-server edge offloading and selection architecture with the advantages of MEC and SWIPT is designed. The MEC server can provide high-speed computing services, but it also has computing costs;
- Under a time-varying environment and the time and energy consumption constraints of the IoT, we propose two non-convex problems related to computing rate and cost. Each non-convex problem is decomposed into two subproblems;
- In contrast to other static optimization method, we propose an AC algorithm of online dynamic optimization by combining the system model. The improved actor module and the critic module are updated iteratively. By the adaptive setting method of k, we can quickly find an offloading scheme that maximizes the computing rate and a selection scheme that minimizes the computing cost.The simulation results verify the effectiveness of the AC scheme.

In Section 2, we review related works. We describe the system model in Section 3. In Section 4, we show the background of the offloading and selection scheme and derive the computing rate and computing cost formulas, respectively.We introduce the mechanism of the deep reinforcement learning(DRL) method in Section 5. The simulation results and detailed analysis are shown in Section 6, and conclusions are drawn in Section 7.

## 2. Related Work

In recent years, edge computing has been extensively studied and implemented in various frameworks. The authors of [9,10,11,12,13,14,15] use the Lyapunov algorithm, heuristic algorithm, and game theory algorithm to solve the edge offloading problem. For example, ref. [9] combines the SWIPT technology to study the edge computing system with a single relay and multiple terminals and used the convex optimization algorithm to obtain the offloading scheme that minimizes the energy consumption. The authors of [10] use two algorithms based on the knapsack problem to optimize the offloading decision in the case of task overflow. The authors of [11] use the Lyapunov optimization algorithm to propose a low-complexity online computing offloading and trajectory scheduling algorithm for Unmanned Aerial Vehicle (UAV) networks to minimize the average energy efficiency. The authors of [12] use SWIPT technology and the Lyapunov optimization framework at the same time to allocate resources to a multi-terminal and single-edge server system, reducing task accumulation and increasing the amount of data processed. The authors of [13] use the Lyapunov algorithm to obtain an offloading policy in edge and cloud networks with the purpose of optimizing energy consumption. The authors of [14] combine the Lyapunov algorithm and energy collection technology to optimize multi-server and multi-terminal model to reduce costs. The authors of [15] focuses on the multi-channel wireless interference environment, designs a distributed offloading algorithm to achieve Nash equilibrium, and obtains the upper limit of the convergence time. These algorithms only obtain the approximate optimal solution according to the current conditions. These algorithms that do not contain a learning mechanism are referred to as static optimization. In static optimization, if the channel or parameters change, the changed parameters will be placed into a large number of formulas and the scheme under new conditions will be obtained by repeated calculations. When the number of terminals is large, we will face the dilemma of complex and huge computing. They cannot interact with the surrounding environment by learning mechanisms or adapt to the ongoing changes in the system quickly.

The way we can interact with the environment by learning is called dynamic optimization. Scheduling methods based on reinforcement learning or deep learning are an important approach for system dynamic optimization [16]. Reinforcement learning can interact with the environment by a trial and correction mechanism in complex and uncertain environments and learn how to achieve goals. Deep learning learns the internal rules of sample data and finds the characteristics of the target using neural networks. The authors of [17,18,19] use deep learning or reinforcement learning to solve the offloading problem under time-varying conditions. For instance, In [17], reinforcement learning is used to obtain a solution that maximizes the profit of the server and the satisfaction perception of the end user. In [18], a three-layer edge computing network is establishedand differential evolution and deep learning are used to jointly optimize UAV deployment and offloading decisions are used to reduce latency. The authors of [19] propose a response time prediction framework based on deep learning to determine whether to offload at nearby edge nodes, which can improve computational offloading performance and select offloading locations. As the scale of the device increases, the environment becomes more complex. The action space of the system expands exponentially. The limited action space of reinforcement learning cannot satisfy the training needs.

The DRL algorithm solves the dimensional disaster of traditional reinforcement learning by using a neural network storage strategy set, which makes it easier to deal with complex environments and realize the optimal processing of edge computing [20]. It is a high-dimensional dynamic optimization method. The authors of [21] use the DRL algorithm to obtain the maximum computing rate and minimum delay. The authors of [22] propose a dynamic framing offloading algorithm based on dual deep Q-networks to make optimal offloading decisions for sequential subtasks of multiple moving vehicles. The authors of [23] combines neural network and Q-learning to reduce the delay and energy consumption of a single server system. We can see that in the above research, most DRL algorithms are applied to ideal multi-terminal systems without considering practical application scenarios such as IoT, microgrids, etc. Only a few papers discuss the existence of multi-server and multi-relay systems. Scholars tend to focus on multi-terminal offloading decisions and ignore the multi-server selection decisions.

In order to solve the above problems, a multi-terminal, multi-relay and multi-server SWIPT system model is designed. This model can comprehensively show the scenario of devices connected to the IoT. Aiming at the problem of edge network computing under time-varying channels and time-varying prices, two non-convex problems are proposed to maximize the computing rate and minimize the computing cost under the constraints of delay and energy consumption. This paper converts every problem into two subproblems. In terms of computing rate, the first subproblem is solved by the actor module, and the AC offloading scheme is obtained. To solve the second subproblem, we allocate a time slot using a convex optimization algorithm to maximize the computing rate in the critic module. For the computing cost, the actor module is used to solve the first subproblem and the AC selection scheme with the smallest computing cost is obtained. Then, we substitute the AC selection scheme into the critic module to solve the second subproblem and calculate the minimum computing cost. Finally, we find the optimal AC offloading algorithm and AC selection algorithm by iterating and updating continuously. In this model, we adopt a binary task offloading policy [7]. All the terminals have two computing states, namely local computing and offloading. On the issue of server selection, we adopt a binary server selection policy that allows for server processing and reject server processing.

## 3. System Model

**Figure 1:**
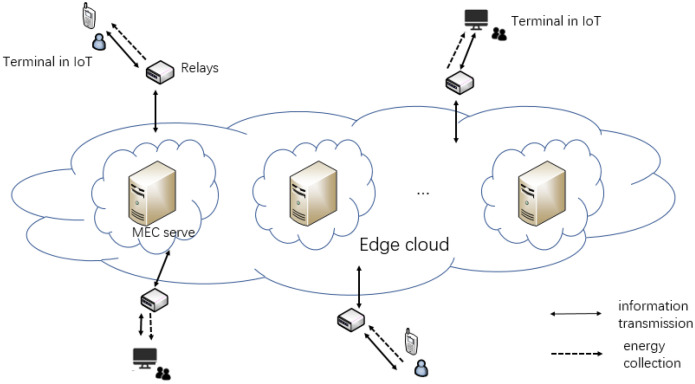
System model.

**Figure 2:**
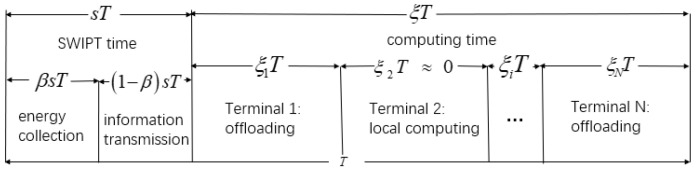
Time frame division.

We have assumed a real situation in IoT. Terminals such as smart phones or computers need the help of edge servers to process data. Users will pay for the offloading as the system cost. We need to maximize the data processing and minimize the cost. The MEC relay system with SWIPT designed in this paper is shown in Figure 1. The system is divided into three parts. The terminals in IoT mean a large number of intelligent terminal devices, which can realize user-oriented data collection, energy collection, and local computing. Multi-relays will extend the transmission range. The part of the edge cloud contains many MEC severs to calculate the offloading task. This paper does not focus on the problem of relay selection. Thus, we assign each terminal a relay to serve it. The system has two time-varying channel gains. $h_{j}$ is defined as a channel gain from the *j*-th server to the relay, and $h_{i}$ is defined as a channel gain from the relay to the *i*-th terminal, *i*∈ (1,2…N), *j*∈ (1,2…M). *N* represents the number of terminals and *M* represents the number of edge servers. The two channel gains remain unchanged in one time frame. However, they vary in the next time frame. The system time is divided into consecutive time frames of equal lengths *T*. We divide *T* into two parts: SWIPT time and computing time. A calculation task needs to be completed in each time frame. The edge servers’ price for processing 1 bit of data $p_{t}^{m}$ is time-varying. The edge server has a stable power supply structure and sends *N* radio frequency (RF) signals. The relay receives the signal, amplifies and forwards it, and then transmits it to *N* terminals. The terminal uses time switching (TS) mode to divide the received RF signals to collect energy and decode information. The collected energy is stored in a rechargeable battery to provide energy for the calculation of the terminal. The decoded information contains servers’ offloading price list within this time frame. We ignore the mutual interference between offloading data and downlink data. Time allocation refers to the reasonable division between SWIPT time sT and computing time ξT in a single time frame. The time allocation in the offloading process is shown in Figure 2.

## 4. Problem Formulation

### 4.1. Offloading and Selection Background

We can use a traversal algorithm to obtain the maximum rate and minimum cost, which is known as the benchmark rate and benchmark cost. However, a traversal algorithm needs to go through $2^{N}$ comparisons and solve problems $2^{N}$ times. Therefore, this paper adopts an online offloading and selection algorithm to obtain offloading and selection actions closer to the benchmark rate and cost without the assumption of knowing future realizations of random channel conditions and data arrivals. In this section, the cost and rate formula is derived and the motivation for choosing this algorithm is explained.

### 4.2. SWIPT Phase

The server transmits a RF signal with normalized power $P_{a}$. Since the transmission power is high and the calculation result of the edge server is usually small, the signal download time and the energy consumption of edge servers are ignored.

Then, the transmitted power at the relay is:

(1) $$P_{r}={k_{r}}^{2}P_{a}{h_{j}}^{2}+{k_{r}}^{2}N_{r},$$

where $N_{r}$ is the noise power at the relay and $k_{r}$ is the amplification and forwarding coefficient of the relay.

The energy collected by the *i*-th device is:

(2) $$E_{i}=\beta P_{r}h_{i}sT$$

where βsT is the time of energy collection, β ∈ (0,1).

### 4.3. Computing Phase

There are two computing modes for all terminals: local computing or offloading. The computing rates in the two states are given below.

#### 4.3.1. Local Computing

Terminals in a local computing state can collect energy and compute tasks at the same time [24]. Therefore, the local computing time is incorporated into the SWIPT time and is ignored in the computing time. $f_{i}$ is the operation frequency. $t_{i}$ is the operation time, $0\leq t_{i}\leq T$. Then $f_{i}t_{i}$ is the operation workload. The total energy consumption of the equipment is [25]:

(3) $$E_{l}oc=k_{i}{f_{i}}^{3}t_{i}$$

where $k_{i}$ is the effective switching capacitance, which depends on the chip structure [26]. There are energy constraints: $E_{l}oc\leq E_{i}$. All the collected energy will be consumed to improve energy efficiency.

The local computing rate $r_{loc}$ (in bits per second) is:

(4) $$r_{loc}=\frac{\sqrt[{3}]{\beta h_{i}s\left({k_{r}}^{2}P_{a}{h_{j}}^{2}+{k_{r}}^{2}N_{r}\right)}}{\sqrt[{3}]{k_{i}}\phi }$$

where ϕ is the number of cycles required to process 1 bit of data.

#### 4.3.2. Offloading Phase

Since the computing rate of the server is much higher than the rate of the terminal, we ignore the time of task calculation and task receiving. In order to improve energy utilization, this section will consume all the collected energy, but the energy consumption cannot exceed the energy collected. Thus, the terminal’s optimal transmit power $p_{i}^{*}$ is:

(5) $$p_{i}^{*}=\frac{E_{i}}{\xi_{i}T}=\frac{\beta h_{i}s\left({k_{r}}^{2}P_{a}{h_{j}}^{2}+{k_{r}}^{2}N_{r}\right)}{\xi_{i}}$$

where $\xi_{i}T$ is the offloading time of the *i*-th terminal. There are time constraints: $s+\sum_{i=1}^{N}\xi_{i}=1$.

It can be deduced that the signal and interference to noise ratio (SINR) in the offloading process is [9]:

(6) $$SINR=\frac{{h_{j}}^{2}{k_{r}}^{2}{h_{i}}^{2}p_{i}^{*}}{{h_{j}}^{2}{k_{r}}^{2}N_{r}+N_{0}}$$

where $N_{0}$ is the noise power at the receiver.

Thus, the offloading computing rate $r_{m}ec$ is:

(7) $$r_{mec}=B\log_{2}\left(1+SINR\right)=B\log_{2}\left(1+\frac{{h_{j}}^{2}{k_{r}}^{2}{h_{i}}^{2}p_{i}^{*}}{{h_{j}}^{2}{k_{r}}^{2}N_{r}+N_{0}}\right)$$

where *B* is the channel bandwidth.

### 4.4. Cost Phase

The total offloading data flow of the system can be expressed as

(8) $$I=r_{mec}\sum_{i=1}^{N}\xi_{i}T$$

The total offloading data flow is evenly divided according to *l*: the number of servers allowed to process. The total cost of the system Ω can be expressed as

(9) $$\Omega =\sum_{j=1}^{N}x_{j}p_{t}^{m}\frac{I}{l}$$

where $x_{i}=1$ indicates that the user allows the *j*-th server to process the offloading information flow. $x_{j}=0$ indicates that the user refuses to process the *j*-th server. $p_{t}^{m}$ is the server’s time-varying price.

### 4.5. Solving Formula

The total computing rate *Q* can be expressed as:

(10) $$Q=\sum_{i=1}^{N}\left(x_{i}r_{mec}+\left(1-x_{i}\right)r_{loc}\right)$$

where $x_{i}=1$ represents the *i*-th terminal in offloading and $x_{i}=0$ represents the *i*-th terminal in local computing.

The problem of the maximum computing rate can be expressed as:

(11) $$\begin{array}{cc} P1:Q^{*}\left(h_{i},h_{j}\right)=maximize\left(Q\right)\;\;\;\; & s.t.\;\left\{\begin{array}{c} s+\sum_{i=1}^{N}\xi_{i}=1\\ s\geq 0,\xi_{i}\geq 0,i\in \left\{0,1\ldots N\right\}\\ x_{i}\in \left\{0,1\right\},i\in \left\{0,1\ldots N\right\} \end{array}\right. \end{array}$$

**Figure 3:**
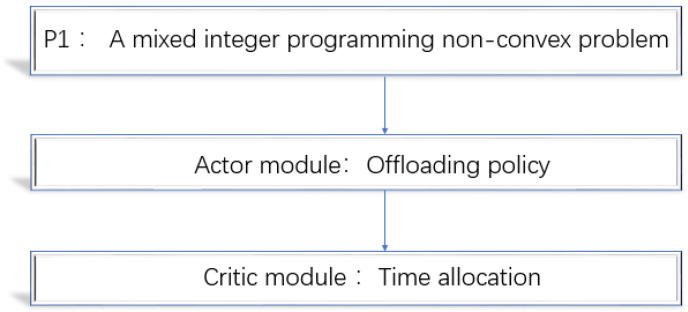
Solving the P1 problem.

The P1 problem involves combinatorial mode selection variable $x_{i}$, which has $2^{N}$ possibilities when it comes to offloading policy. Therefore, the P1 problem is a hard mixed integer programming non-convex problem and is difficult to address. However, we observed that two variables of $a,\xi_{i}$ are jointly concave. Once offloading policy $x_{i}$ is given, the P1 problem can be transformed into a convex problem, P2, which concerns time allocation. In order to solve the P1 problem, this paper converts the P1 problem into two subproblems: offloading policy and time allocation [21], as shown in Figure 3.

What is desired is an offloading scheme close to the benchmark rate. That is, in a time-varying IoT, we need to design a policy function that will be updated gradually to take action quickly and accurately. Therefore, we choose the online algorithm based on the policy gradient. The deep neural networks (DNN) structure is simple, but it can gradually improve the policy function from experience to solve complex decision-making problems. It is suitable for a changeable and unknown IoT. Therefore, we take DNN as the actor module to optimize the network parameters for the purpose of maximizing the rate. The offloading policy part uses the actor module to find the AC offloading scheme. This will be explained in detail in Section 5.1.

(12) $$\begin{array}{cc} P2:Q^{*}\left(h_{i},h_{j},x_{i}^{*}\right)=maximize\left(Q\right)\;\;\;\; & s.t.\;\left\{\begin{array}{c} s+\sum_{i=1}^{N}\xi_{i}=1\\ s\geq 0,\xi_{i}\geq 0,i\in \left\{0,1\ldots N\right\} \end{array}\right. \end{array}$$

After the AC offloading scheme is obtained by the actor module, P1 becomes a solvable convex problem P2. At the same time, we need a standard to judge whether the action is good or bad. Therefore, we use the convex optimization algorithm of one-dimensional double-section search [27] to allocate the SWIPT time and the offloading time effectively and solve the P2 problem to measure the performance. We call it a critic module. The critic module we use only needs to analyze and solve the optimal time allocation problem to evaluate accurately. Then, a small amount of comparison can complete the optimization goal. Compared with a traditional critic module, there is no need to compare many value functions, and the small error brought by the value function does not affect the system. The two modules obtained repeatedly and alternately to adapt to environmental changes are called an AC offloading algorithm.

The problem of minimum computing cost can be expressed as:

(13) $$\begin{array}{cc} P3:\Omega^{*}\left(h_{i},h_{j},p_{t}^{m},I\right)=maximize\left(\Omega \right)\;\;\;\; & s.t.\;\left\{\begin{array}{c} l>0,p_{t}^{m}>0\\ s\geq 0,\xi_{i}\geq 0,i\in \left\{0,1\ldots N\right\}\\ x_{j}\in \left\{0,1\right\},j\in \left\{0,1\ldots M\right\} \end{array}\right. \end{array}$$

**Figure 4:**
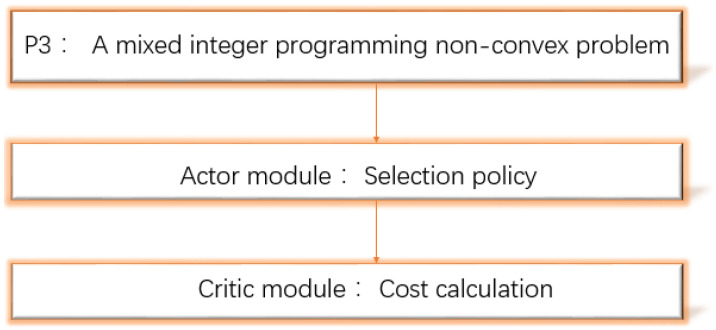
Solving the P3 problem.

Combinatorial mode selection variable $x_{j}$ has $2^{M}$ possibilities for selection policy. Thus, P3 is also a mixed integer programming non-convex problem. We also decomposes p3 into two parts: selection policy and cost calculation, as shown in Figure 4. What we want is a selection policy close to the benchmark cost. Similar to the offloading scheme, we take the other DNN neural network as the actor module to optimize the network parameters with the goal of minimizing the cost. In the selection policy, the actor module is used to find the AC selection scheme. This will be described in detail in Section 5.1.

After the AC selection scheme is obtained via an actor module, P3 becomes the solvable problem P4. We substitute the AC selection scheme into the P4 problem to calculate the cost to judge the quality of the scheme. We call it the critic module. The critic module we use only needs to analyze and solve the cost problem to make an accurate evaluation.Then, a small number of comparisons can complete the optimization goal. Compared with traditional critic module, there is no need to compare many value functions, and the small error brought by the value function does not affect the system. The two modules obtained repeatedly and alternately to adapt to the change in the environment are called the AC selection algorithm. The common use of the actor module and the critic module makes the updating of the actions of the edge system more accurate and faster than other DRL methods.

(14) $$\begin{array}{cc} P4:\Omega^{*}\left(h_{i},h_{j},p_{t}^{m},I,x_{j}^{*}\right)=maximize\left(\Omega \right)\;\;\;\; & s.t.\;\left\{\begin{array}{c} l>0,p_{t}^{m}>0\\ s\geq 0,\xi_{i}\geq 0,i\in \left\{0,1\ldots N\right\} \end{array}\right. \end{array}$$

## 5. Ac Algorithm

AC algorithms perform transitions from input states to output actions in uncertain edge environments [28]. The traditional actor module is used to deal with continuous actions. In order to meet the actions of binary decision, we quantify the relaxed actions. In this paper, the modified actor module is used to obtain (input state and output action) pairs. Compared with the traditional critic module based on the value function network [29], we choose to improve it because the parameters in the value function update process may affect the performance. The improved critic module accurately evaluates the actions taken by the actor module and trains the DNN by using the system information to solve the objective function. To reduce data dependencies, we added replay memory. We store pairs in the replay memory and continuously extract training samples from the replay memory; 80% of the data in the sample are used for training and 20% for testing. Compared with the traditional actor-critic structure, this method combines the specific system information to make the DRL training process more robust and converge faster. We train the neural network to evaluate the actions taken by the actor module by using the objective function. The parameters of the neural network are updated to generate more accurate strategies. Due to the different input states and enhancement purposes of offloading and selection schemes, different AC architectures need to be used for applied research. At the same time, we list the structure of the DQN algorithm updated based on the value function and make a simulation comparison in Section 6.

### 5.1. Application of the Offloading Scheme

**Figure 5:**
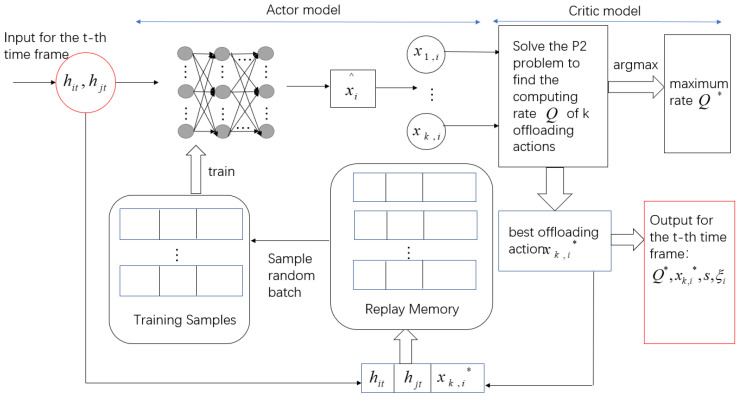
AC algorithm structure of offloading scheme.

Two time-varying channel gains are used as the input of the offloading scheme in the *t*-th time frame. The purpose is to find the offloading action that maximizes the computing rate of the system. Therefore, the algorithm structure is shown in Figure 5.

We input two channel gains at the *t*-th time frame and get the relaxed offloading action $\hat{x_{i}}$ when using an actor module.

The activation function of the hidden layer is set to Relu, and the activation function of the output layer is set to Sigmoid. The number of neurons in the input and output layers is *N*. Quantize $\hat{x_{i}}$ to obtain *k* quantized action sets $x_{k,i}$, $i\in \left\{0,1\ldots N\right\}$, which controls the computing state of the *i*-th terminal. In general, *k* can be any integer within [1, $2^{N}$]. A larger *k* has a better solution quality and higher computational complexity, and vice versa.

To balance performance and complexity, we adopt the order-preserving quantization method [21] and the adaptive setting method of *k*. The order-preserving quantization method adjusts the relaxed offloading actions into *k* quantized action sets according to the formula [21]

(15) $$\begin{array}{c} x_{1,i}=\;\left\{\begin{array}{c} 1,\hat{x_{i}}>0.5\\ 0,\hat{x_{i}}\leq 0.5 \end{array}\right. \end{array}$$

(16) $$\begin{array}{c} x_{k,i}=\;\left\{\begin{array}{c} 1,\hat{x_{k,i}}>\hat{x_{k-1,i}}\\ 1,\hat{x_{k,i}}=\hat{x_{k-1,i}}\;\mathrm{and}\;\hat{x_{k-1,i}}\leq 0.5\\ 0,\hat{x_{k,i}}=\hat{x_{k-1,i}}\;\mathrm{and}\;\hat{x_{k-1,i}}>0.5\\ 0,\hat{x_{k,i}}<\hat{x_{k-1,i}} \end{array}\right. \end{array}$$

When the neural network gradually explores the optimal policy over time, with a certain update interval Λ, the value of *k* is optimized according to the formula [21]

(17) $$\begin{array}{c} k=\;\left\{\begin{array}{c} N,t=1\\ min(max\left(k_{t-1}^{*},k_{t-2}^{*},\ldots ,k_{t-\Lambda }^{*}\right)),tmod\Lambda =0\\ k_{t-1},otherwise \end{array}\right. \end{array}$$

$k_{t}^{*}$ denotes the ranking of the optimal offloading action among the *k* quantified actions sets.

In the critic model, we substitute *k* quantized action sets $x_{k,i}$ to solve the P2 convex problem and the corresponding data $Q,s,\xi_{i}$ are calculated. The maximum computing rate $Q^{*}$ and the corresponding optimal offloading action $x_{k,i}^{*}$ are stored in the replay memory in the form of $\left(\langle h_{it},h_{jt}\rangle ,x_{k,i}^{*}\right)$ to train the actor model. $s,\xi_{i}$ represent the time allocation for the corresponding decision.

The replay memory has a limited capacity. When the memory is full in the *t*-th time frame, the newly generated data pair is stored and the old data pair is discarded. A batch of training samples $\left(\langle h_{i\rho },h_{j\rho }\rangle ,x_{\rho }^{*}\right),\rho \in T_{t}$ are randomly sampled from the memory using the experience replay technique. $T_{t}$ is the time index of the sampling sample. The DNN is trained every σ time frame, and the parameters $\omega_{it}$ of DNN are continuously updated to generate a new and accurate offloading policy. $\omega_{it}$ updates by applying the Adam algorithm [30]. The loss function is expressed as:

(18) $$L\left(\omega_{it}\right)=-\frac{1}{\left|T_{t}\right|}\sum_{\rho \in T_{t}}\left\{{\left(x_{\rho }^{*}\right)}^{⊺}log_{2}f_{\omega_{it}}\left(h_{i\rho },h_{j\rho }\right)+{\left(1-x_{\rho }^{*}\right)}^{⊺}log_{2}\left[1-f_{\omega_{it}}\left(h_{i\rho },h_{j\rho }\right)\right]\right\}$$

where $\left|T_{t}\right|$ denotes the size of the index and the superscript ${}^{⊺}$ denotes the transpose operator.

**Figure 6:**
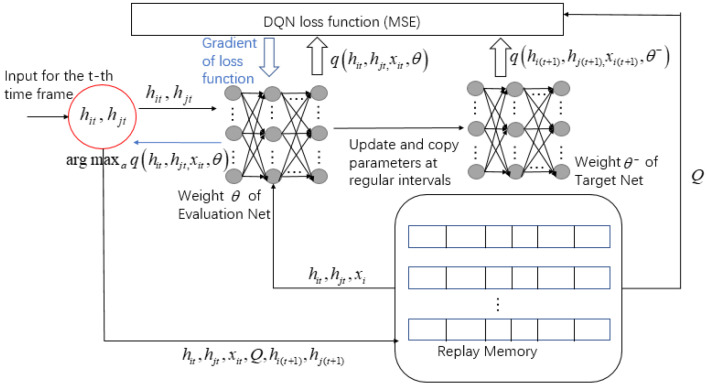
DQN algorithm of offloading scheme.

For the comparison of algorithm effectiveness in Section 6, the specific steps of the DQN algorithm of the offloading scheme are plotted in Figure 6. Since the DQN algorithm is not the focus of this article, we will not go into detail here.

### 5.2. Application of the Selection Scheme

**Figure 7:**
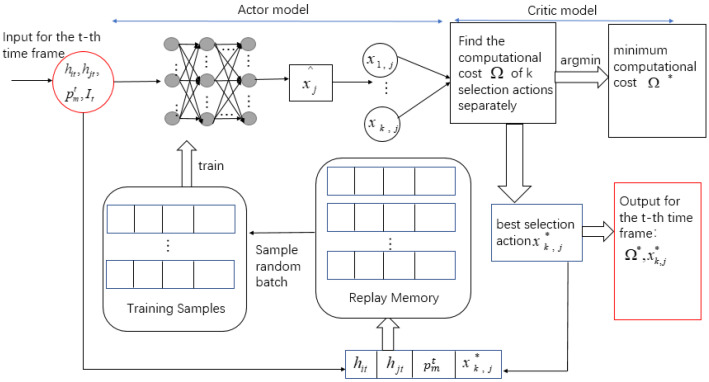
AC algorithm structure of selection scheme.

The inputs of the selection scheme are two time-varying channel gains, the server’s time-varying price, and the total offloading data flow. The purpose is to find the selection action that minimizes the cost. Therefore, the algorithm structure is shown in Figure 7.

**Figure 8:**
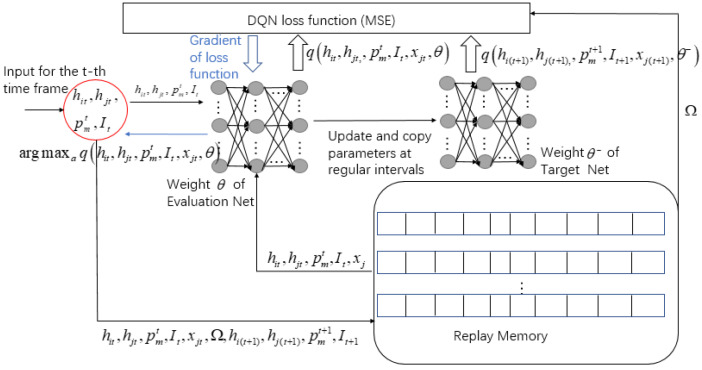
DQN algorithm of selection scheme.

For the comparison of algorithm effectiveness later in the article, the specific steps of the DQN algorithm of the selection scheme are plotted in Figure 8. Because the DQN algorithm is not the focus of this article, we will not go into detail here.

We use the method in Section 5.1 to get $x_{k,j}$ using the actor module. We obtain the minimum computing cost $\Omega^{*}$ and the corresponding optimal selection action $x_{k,j}^{*}$ by training the critic module and then store them in the replay memory in the form of $\left(\langle h_{it},h_{jt},p_{t}^{m},I\rangle ,x_{k,j}^{*}\right)$. A batch of training samples $\left(\langle h_{i\vartheta },h_{j\vartheta },p_{t}^{m},I\rangle ,x_{\vartheta }^{*}\right),\vartheta \in T_{t}$ is randomly sampled from the memory using the experience replay technique. The parameter $\omega_{jt}$ of DNN is continuously updated to generate new and accurate selection policies. $\omega_{jt}$ is updated by applying the Adam algorithm [30]. The loss function is denoted as:

(19) $$L\left(\omega_{jt}\right)=-\frac{1}{\left|T_{t}\right|}\sum_{\vartheta \in T_{t}}\left\{{\left(x_{\vartheta }^{*}\right)}^{⊺}log_{2}f_{\omega_{jt}}\left(h_{i\vartheta },h_{j\vartheta },p_{t}^{m},I\right)+{\left(1-x_{\vartheta }^{*}\right)}^{⊺}log_{2}\left[1-f_{\omega_{jt}}\left(h_{i\vartheta },h_{j\vartheta },p_{t}^{m},I\right)\right]\right\}$$

In the proposed time-varying environment, the modified AC algorithm reduces the correlation of the data using experience replay technology. It makes full use of the system information to obtain the evaluation of the action accurately and reduces the influence of neural network parameters on the action policy. The number of comparison actions is reduced by the adaptive setting method of K. Compared with the traditional actor-critic structure, this method combines specific system information, makes the DRL training process more robust, and speeds up convergence.

## 6. Simulation Analysis

**Figure 9:**
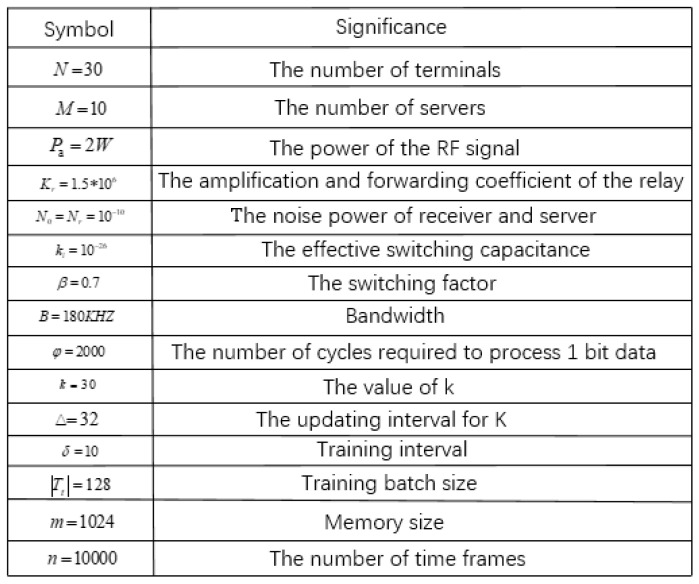
Simulation values.

In this section, we evaluate the performance of the proposed offloading and selection schemes using simulations. The specific simulation data are shown in Figure 9. In the real scene, single server covers a wide range and connects many terminals. However, in order to facilitate the comparison with the traversal algorithm, the number of terminals is set to 30. In order to increase the selectivity of servers, we set up 10 servers. At the same time, the power of RF signal, bandwidth, and noise power match the settings of the IoT. These simulated data will help the algorithm simulation and be closer to a real IoT scenario.The training parameters of the DQN algorithm and AC algorithm are consistent. Both algorithms extract data randomly, but the AC algorithm updates in one step while the DQN algorithm updates every 50 steps.

To better showcase the performance, this paper adopts the normalized ratio comparison method:

(20) $$\tilde{Q}=\frac{\bar{Q}}{\hat{Q}},\tilde{\Omega }=\frac{\bar{\Omega }}{\hat{\Omega }}$$

where $\bar{Q},\bar{\Omega }$ represent the maximum computing rate and the minimum computing cost obtained by different schemes, respectively. $\hat{Q},\hat{\Omega }$ represent the benchmark rate and the benchmark cost, respectively, $\tilde{Q}\in \left(0,1\right),\tilde{\Omega }\geq 1$.

### 6.1. Comparison of Computing Rates for Adding Relay

The existence of the relay resists the channel fading caused by long-distance transmission and expands the communication range. In this paper, a single relay is added to the edge computing network. We observe the impact on the system rate.

**Figure 10:**
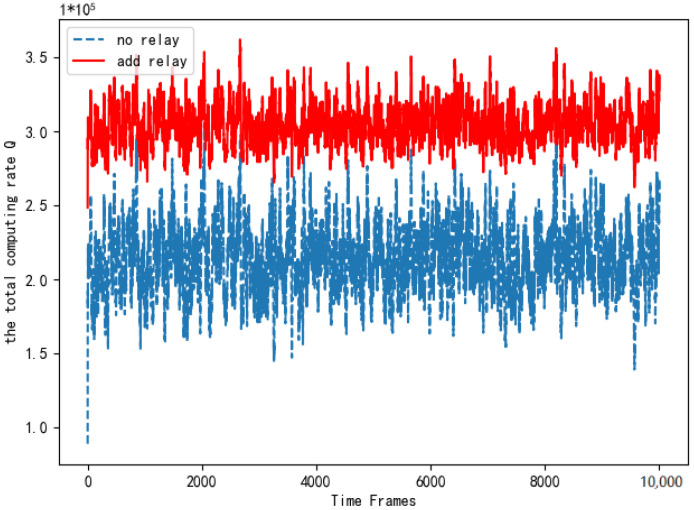
Comparison of total computing rate when adding relays.

Figure 10 compares the total computing rate of the system with the relay and the system without the relay. As is shown in Figure 10, the total computing rate after adding the relay is better than that without the relay. The simulation verifies that adding the relay not only expands the communication range but also improves the computing rate of the system significantly. It proves the necessity of adding relay equipment.

### 6.2. Performance Analysis of Offloading Scheme

#### 6.2.1. Influence of Neural Network Parameters on Offloading Scheme

In this paper, DNN plays a very important role in obtaining the optimal offloading scheme. The best parameter settings are obtained using a simulation comparison.

**Figure 11:**
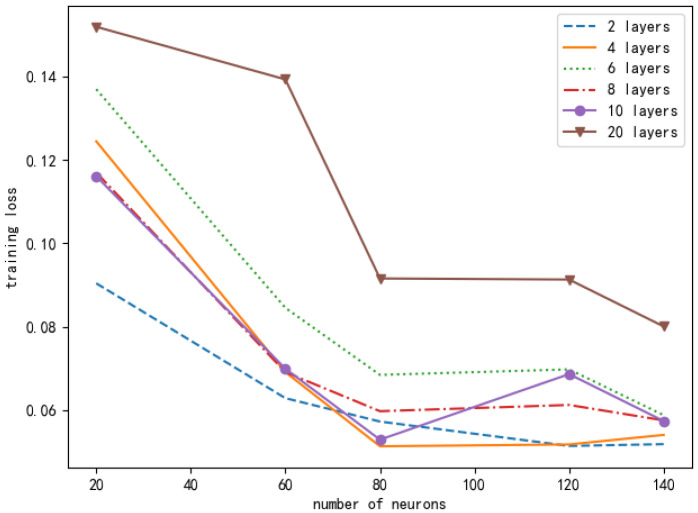
Comparison of loss function values under different numbers of neurons and different neural network layers in a DNN.

**Figure 12:**
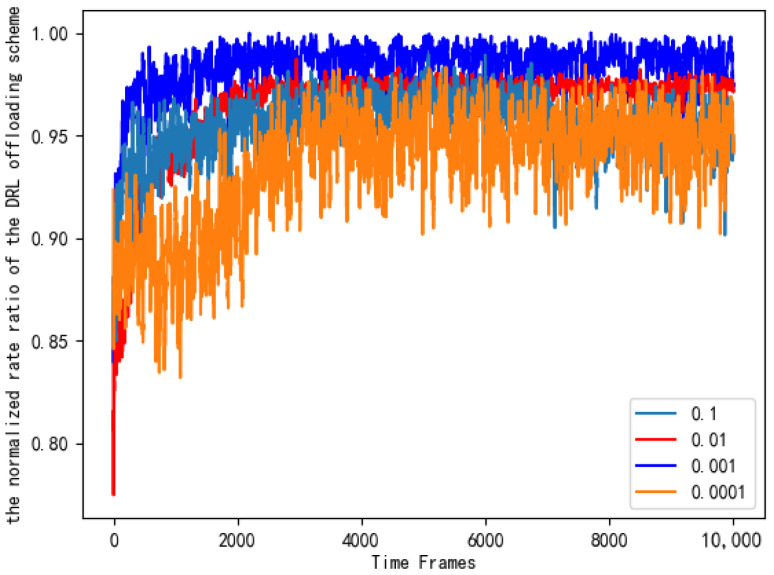
Normalized rate ratios of AC offloading schemes with different learning rates.

It can be seen from Figure 11 that the number of layers and neurons of different neural networks will cause different training losses. In order to ensure the accuracy of the experimental results, we conducted the same simulation ten times, and the data in the figure is the average value. Based on the principle of selecting small and stable training losses, we chose a DNN composed of one input layer, three hidden layers, and one output layer. Each hidden layer contains 80 neurons. Figure 12 looks at the different learning rates of the neural networks and compares their normalized rate ratio to the AC offloading scheme. As can be seen from Figure 12, if the learning rate is set too high, the network will fail to converge. If the learning rate is set too low, the network will converge very slowly, which will increase the time to find the optimal value. When the learning rate is 0.001, the performance of the AC offloading scheme is significantly better, and it is closer to the benchmark offloading scheme.

#### 6.2.2. Ac Offloading Scheme Performance

First, we need to verify the effectiveness of the DNN.

**Figure 13:**
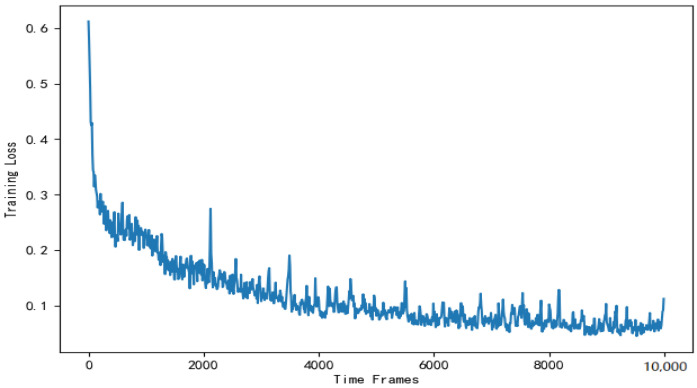
Training loss of the DNN used to solve the offloading scheme.

Figure 13 shows the training loss of DNN in the actor module. As is shown in Figure 13, the training loss gradually decreases and stabilizes around t=4000. There are fluctuations in the loss values in the graph. This is mainly due to the random sampling of training data and does not affect the DNN’s effectiveness.

**Figure 14:**
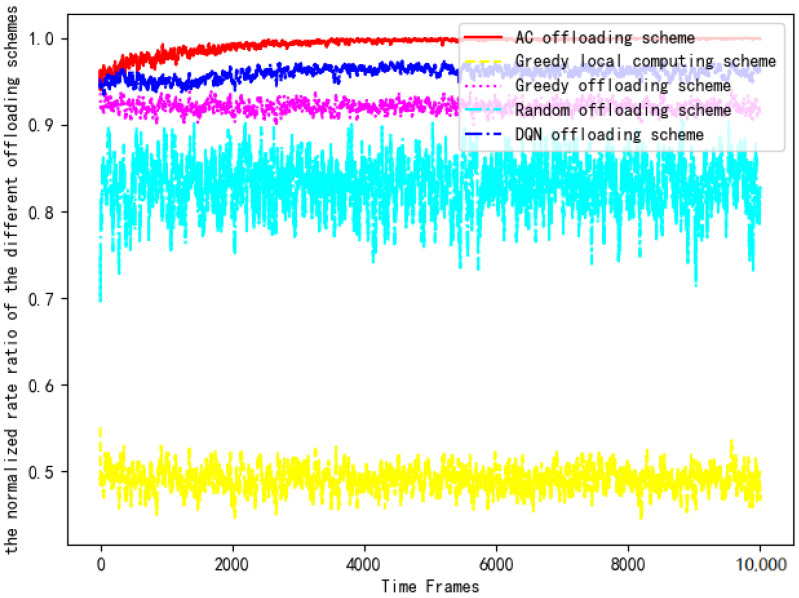
Comparison of the normalized rates of each scheme.

Then, the computing rates of five offloading schemes are substituted into Equation (14) for normalized ratio rate comparison. Figure 14 compares the computing rate of the AC offloading scheme, the DQN offloading scheme, the greedy offloading scheme, the greedy local computing scheme and the random offloading scheme with the benchmark rate.

Greedy local computing scheme: each terminal only completes the computing task using local computing. The offloading scheme is fixed as [0, 0, 0, 0, 0, 0, 0, 0, 0, 0].

Greedy offloading scheme: each terminal only completes the computing task by offloading. The offloading scheme is fixed as [1, 1, 1, 1, 1, 1, 1, 1, 1, 1].

Random offloading scheme: Each terminal chooses to complete the task by using local computing or offloading randomly.

AC offloading scheme: Each terminal chooses to complete the task by local computing or offloading by training the AC algorithm. The goal of each terminal is to maximize the computing rate.

DQN offloading scheme: Each terminal chooses to complete the task by local computing or offloading by training the DQN algorithm. The goal of each terminal is also to maximize the computing rate.

It can be clearly seen that the AC offloading scheme is significantly better than the other schemes in Figure 14. The computing rate of the AC scheme in the early stage is similar to the DQN offloading scheme and the greedy offloading scheme, but as the DNN is trained, the normalized rate value quickly reaches the optimal level and maintains a stable trend. Due to the use of the DRL algorithm to interact with the environment using a trial-and-error mechanism in a complex and uncertain environment, the AC offloading scheme and the DQN offloading scheme have an obvious performance advantage in the later stages. However, because the q-value influenced by the parameters of the neural network is contained in the value function of the DQN algorithm, the DQN offloading scheme fails to achieve superior performance that is closer to the benchmark scheme. In addition, the computing power of terminal devices in IoT is not sufficient to process all the data. The computing rate of the greedy local computing scheme is the lowest, only reaching 50% of the performance of the AC offloading scheme. The computing rate of the random offloading scheme is higher than that of the greedy local computation scheme because offloading decisions in stochastic policy often change in a random manner, which results in a greater chance of approaching the optimal solution. The greedy offloading scheme offloads all tasks to the edge server for computing, which makes full use of the technical advantages of edge technology but ignores the computing power of the device. Finally, the normalized rate values of the greedy offloading scheme, the greedy local computing scheme, and the random offloading scheme are all lower than 0.95 due to the lack of reasonable scheduling of the computing power of edge servers and terminal devices. Therefore, the above three offloading schemes are not recommended.

### 6.3. Performance Analysis of Selection Scheme

#### 6.3.1. Influence of Neural Network Parameters on Selection Scheme

**Figure 15:**
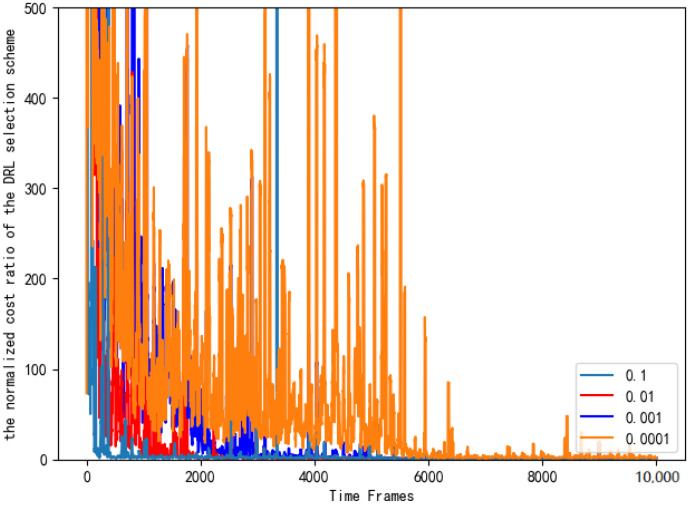
Normalized cost ratios of AC selection schemes with different learning rates.

Figure 15 sets the different learning rates of the neural network and compares the normalized cost ratio of the AC selection schemes.As can be seen from Figure 15, when the learning rate is 0.1, the performance of the AC selection scheme is significantly better, and it is closer to the benchmark selection scheme.

#### 6.3.2. Ac Selection Scheme Performance

First, we need to verify the effectiveness of the DNN.

**Figure 16:**
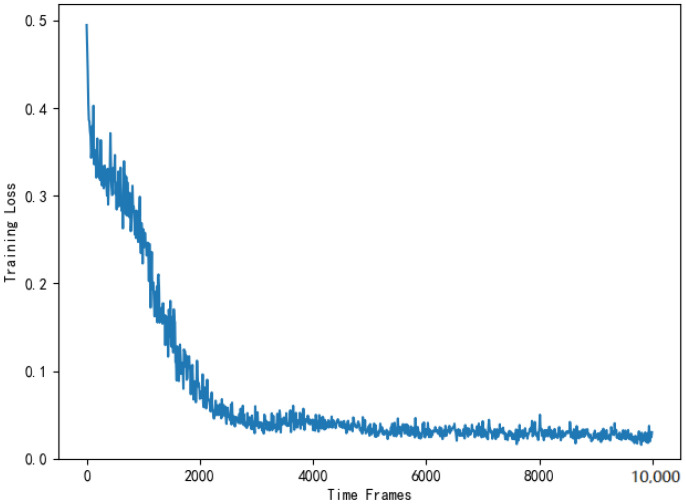
Training loss of the DNN used to solve the selection scheme.

**Figure 17:**
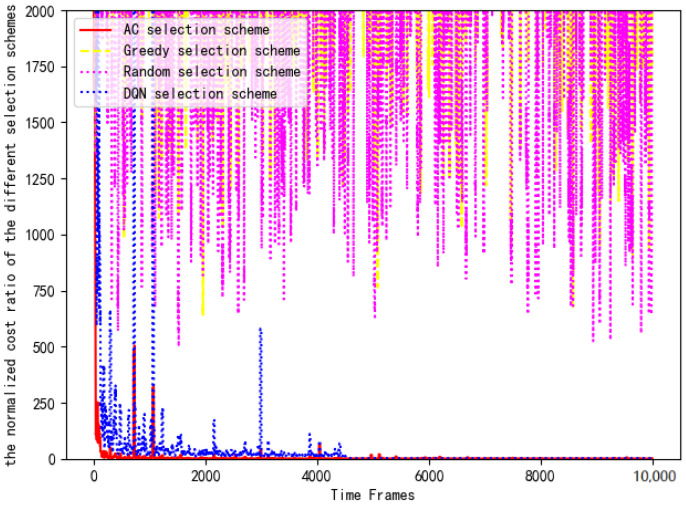
Comparison of normalized costs of each scheme.

Figure 16 shows the training loss of DNN in the actor module of the AC selection scheme. As is shown in Figure 16, the training loss gradually decreases and stabilizes around t=4000. The reason for the fluctuation is consistent with the offloading scheme. Then, the computing costs of four selection schemes are substituted into Equation (14) for normalized cost ratio comparison. Figure 17 compares the computing cost of the AC selection scheme, the DQN selection scheme, the greedy selection scheme and the random selection scheme with their respective benchmark costs.

Greedy selection scheme: The offloading data stream is equally distributed to each server for calculation processing, and the selection scheme is fixed as [1, 1, 1, 1, 1, 1, 1, 1, 1, 1].

Random selection scheme: The offloaded data stream is randomly distributed. There is no case where the selection scheme is [0, 0, 0, 0, 0, 0, 0, 0, 0, 0]. We must select at least one server for data processing.

AC selection scheme: We control servers to offload or not to offload by training the AC algorithm. The goal of each terminal is to minimize the computing cost.

DQN selection scheme: We control servers to offload or not to offload by training the DQN algorithm. The goal of each terminal is also to minimize the computing cost.

It can be clearly seen that the AC selection scheme and the DQN selection scheme are significantly better than the other schemes in Figure 17. In the early stage, the costs of the AC selection scheme and the DQN selection scheme are 2000 times that of the benchmark scheme. With the training of the DRL algorithm, the AC selection scheme and the DQN selection scheme quickly reach the optimal cost and maintain long-term stability. However, it can be seen that the computing cost of the DQN selection scheme is higher than that of the AC selection scheme because the former uses the q-value function in the algorithm iteration. Compared with the greedy selection scheme, the random selection scheme is better due to its randomness. However, there is still a significant performance gap compared with the AC option and DQN option. Because the time-varying price factor is not considered, the cost fluctuations of the greedy selection scheme and random selection scheme are about 1000–2000 times that of the benchmark scheme. Compared with the benchmark scheme, its consumption cost is too large, so it is not recommended.

Compared with the traditional traversal algorithm, the program execution delay of the AC algorithm and DQN algorithm is reduced significantly in Table 1. Because the offloading scheme needs to control 30 terminal devices and the selection scheme will dominate 10 edge servers, the program execution delay of the offloading algorithm is greater than that of the selection. Even in the case of 30 terminals and 10 servers, the program execution delay of the DQN algorithm and AC algorithm is lower than that of the traversal algorithm due to their learning ability. However, in the face of multiple terminals in the IoT, the DQN algorithm has the property of exhaustive search in iteration, which is not suitable for high-dimensional action space. When the number of terminals is 30, the program execution delay is longer than that of the AC algorithm. The AC algorithm has the fewest program execution delays of selection and offloading among the three algorithms. This proves that the AC algorithm has good performance in the selection and offloading schemes. The AC algorithm achieves a near-optimal computing cost and computing rate while reducing operation delay. Therefore, the AC scheme in this paper has certain practical value.

## 7. Conclusions

This paper combines two advanced technologies, MEC and SWIPT, to simulate a multi-relay, multi-terminal, and multi-server SWIPT edge computing model. Firstly, we derive the system computing rate and computing cost. Secondly, under the time-varying price of edge server processing data and two time-varying channels, a DRL algorithms is used to obtain an offloading scheme and selection scheme that maximize the computing rate and minimize the computing cost. Finally, we come to a conclusion. We can obtain learning results close to the benchmark rate and benchmark cost in a shorter time when using DRL algorithm. Compared with the traversal algorithm, the AC algorithm can achieve the same excellent performance and reduce the program execution delay greatly. Due to the value function network parameters and exhaustive search when selecting actions, the performance and program execution delay of the DQN algorithm are slightly worse than that of the AC algorithm, but it still has advantages compared with other algorithms. The superiority of the DRL algorithm is verified. In the future, we think edge cache technology will also be a good choice. It can store the resources that are often used in the memory of the device or server, which will send data quickly and reduce the computing cost when the terminals need them. At the same time, we will continue to pay attention to the application of the DRL algorithm.

## Figures and Tables

**Table 1:** Comparison of the average program execution delay per time frame of different algorithms for selection.

	AC Algorithm	DQN Algorithm	Traversal Algorithm
offloading delay(s)	0.0548	0.1035	3.526
selection delay(s)	0.0073	0.0102	0.196

## Data Availability

Not applicable.
